# Characteristic wave detection in ECG signal using morphological transform

**DOI:** 10.1186/1471-2261-5-28

**Published:** 2005-09-20

**Authors:** Yan Sun, Kap Luk Chan, Shankar Muthu Krishnan

**Affiliations:** 1 Bioinformatics Institute, Singapore 138671; 2 Biomedical Engineering Research Center, School of Electrical and Electronic Engineering, Nanyang Technological University, Singapore 639798

## Abstract

**Background:**

Detection of characteristic waves, such as QRS complex, P wave and T wave, is one of the essential tasks in the cardiovascular arrhythmia recognition from Electrocardiogram (ECG).

**Methods:**

A multiscale morphological derivative (MMD) transform-based singularity detector, is developed for the detection of fiducial points in ECG signal, where these points are related to the characteristic waves such as the QRS complex, P wave and T wave. The MMD detector is constructed by substituting the conventional derivative with a multiscale morphological derivative.

**Results:**

We demonstrated through experiments that the Q wave, R peak, S wave, the onsets and offsets of the P wave and T wave could be reliably detected in the multiscale space by the MMD detector. Compared with the results obtained via with wavelet transform-based and adaptive thresholding-based techniques, an overall better performance by the MMD method was observed.

**Conclusion:**

The developed MMD method exhibits good potentials for automated ECG signal analysis and cardiovascular arrhythmia recognition.

## Background

The detection of the major characteristic waves in ECG, namely the QRS complexes, P and T waves, is one of the essential tasks in ECG analysis. The performance of an automated ECG analysis system depends heavily on the reliable detection of these fiducial waves. The difficulties of characteristic waves detection lie in oscillations in the baseline, irregular morphology of the waveforms, and frequency overlapping among the wide-band distribution of the characteristic waves [1], etc.

A significant amount of research effort has been devoted to the automated detection of the fiducial (reference) points of the ECG characteristic waves [2-12]. Most of these methods are filtering or adaptive thresholding based, which exhibit limitation in real application. Very few algorithms work well for the detection of all fiducial points such as the onsets and offsets of the P wave, T wave and the QRS complex (also known as the ECG wave boundaries). The main drawback of filtering-based approach is that frequency variations in the characteristic waves often adversely affect its performance. The frequency distribution of QRS complexes generally overlaps with that of the noise, resulting in both false positive and false negative detections. The main problems of the thresholding techniques are their high noise sensitivity and their low efficiency when dealing with odd morphologies. Therefore, more sophisticated signal processing techniques are needed to facilitate the development of new detection schemes with higher detection accuracy.

As a nonlinear filtering technique, it has been proven that morphological dilation and erosion satisfy the causality and the additive semigroup property required by multiscale analysis [13-15] for signals of any dimension with local maxima and local minima as singular points. The fiducial points in ECG signal, such as the Q wave, R peak, S wave, the onsets and offsets of the P wave and T wave, can be regarded as such singular points [16,17]. In this paper, a new multiscale morphological derivative (MMD) transform-based technique, was developed for the detection of the fiducial points of the ECG characteristic waves. By applying a morphological derivative transform defined at different scales, noise sensitivity inherent in single scale operation can be reduced in MMD method. In addition, the problem of position deviation existed in wavelet transform-based techniques [10] can be avoided due to the nonlinearity of morphological transform. As a result, tracing across scales to locate the singular points is not needed.

### The proposed multiscale morphological derivative (MMD) detector

In the present study, the signal to be processed is limited to continuous function *f *: ^2 ^→  with only finite oscillations on a closed interval which is differentiable everywhere except at some singular points. A singular point in the one-dimensional signal is defined as a point with derivative on the right and the derivative on the left exist with different signs.

For the singular point to be defined using multiscale morphological derivative, the derivative on the right can be represented by morphological sup-derivative , which is defined as



Similarly, the derivative on the left can be represented by morphological inf-derivative , which is defined as



Here, the notation of [18] is used to introduce morphological operators on functions. Denoting the functions, *f *: *D *⊂ ^*n *^→  and *g*_*s *_: *G*_*s *_⊂ ^*n *^→  (*s *> 0), the two fundamental operations of multiscale morphology are:





where *D*_*x *_is the translation of *D*, *D*_*x *_= *x *+ *t*:*t ∈ D*, sup(*f*) and inf (*f*) refer to the supremum (least upper bound) and infimum (greatest lower bound) of *f*, *s *is scale, and *g*_*s *_is the scaled structuring function [19]. In the discrete case where the function is a finite set of points, max() and min() are used instead of sup() and inf().

We propose a multiscale morphological derivative difference , to be defined to characterize the difference between the left and right derivatives as follows:



The scaled version of  at scale *s*, , can then be defined as:



By choosing a flat structuring function, where *g*_*s*_(*x*) = 0, *x *∈ *G*, where *G *= {*x*: ||*x*|| ≤ *s*} [14], the above multiscale morphological derivative transform described by Equation (6) is simplified to the following process: Choose a moving window with a length of (2*s *+ 1) samples and find the maximum and minimum values in the window, as well as the value of the signal at the cental point *f*(*x*). Then, the MMD transform at the central point can be specified as



At a positive peak in ECG signal, its left derivative is positive and its right derivative is negative, therefore, positive peaks in the ECG signal correspond to the local minima in . At the onset or offset of a positive peak, there is an abrupt increase in its derivative value from left to right. So, the onsets and offsets correspond to the local minima in . As applied to ECG lead II signal, the R peak, Q wave an S wave correspond to the local minima of the , while the onsets and offsets of the P wave and T wave correspond to the local maxima of the . Hence the characteristic QRS complex, P wave and T wave, can be detected using the proposed MMD detector by detecting the local extrema in the MMD transformed signal.

### Characteristic wave detection in ECG using the MMD detector

The MMD detector is a single lead detection method. In this paper, we only use the ECG lead II for algorithm development and testing. A similar analysis can be done to extend method to other leads. The detailed procedure for ECG characteristic wave detection using the proposed MMD detector, is described as follows:

1. ECG signal is preprocessed by morphological filtering for noise reduction and baseline correction.

2. Multiscale morphological transform is performed on the preprocessed input signal.

3. The local maxima and minima with absolute amplitude larger than a threshold, *Th*_*f*_, at a selected scale *s*_*m *_are detected (*s*_*m *_= 20 for MIT-BIH database and *s*_*m *_= 15 for QT database in this study). The local minima with absolute amplitude larger than a threshold, *Th*_*R*_, are detected as R peaks, where, the selection of *Th*_*R *_and *Th*_*f *_is based on an adaptive thresholding from the histogram of the MMD transformed data.

4. For each detected R peak, the first local maximum point on its left is detected as the beginning of the R wave; the first maximum point on its right side is detected as the end of the R wave.

5. The first local minimum from the left of the positive R wave is detected as the Q wave. If the minimum cannot be detected, the Q wave is judged to be missing. (There is a time interval for Q wave detection, which is set as the prior clinical value of QRS complex, here, 0.12*s*).

6. The first local minimum from the right of the positive R wave is detected as the S wave. Otherwise, the S wave is judged to be missing. Same time interval as for Q wave detection is set for S wave detection.

7. The subsequent two consecutive local maxima from the left of the Q wave are detected as the offset and onset of the P wave; the first and second local maxima from the right of the Q wave are detected as the onset and offset of the T wave, respectively.

The preprocessing in step 1 is performed as follows:



where *f_o _*is the original input signal; *f*_*b *_is the baseline drift signal; *f *is the signal after preprocessing; o is morphological opening operator; • is morphological closing operator; structuring elements, *B*_*o*_, *B*_*c *_and *B*, are selected based on the properties of ECG characteristic waves. Further details can be found in [20]. For each preprocessed signal, its morphological derivative at scale *s*_*m *_was calculated according to Equation (7) and its local maxima and minima were detected. It is known that the number of maxima or minima at a larger scale is much less than that at a lower scale. In addition, high frequency noise decays greatly at large scales so that less extrema due to noise are found at larger scale. Therefore, morphological derivative transformed signal at a larger scale was used for detecting the locations of the objective feature points. However, in order not to smooth the characteristic waves in ECG, *s*_*m *_should be as large as possible but less than *W*_*w*_*f*_*s*_, where *W*_*w *_is the width of the characteristic wave, and *f*_*s *_is the sampling frequency of ECG signal. The width of QRS is generally from 0.06*s *to 0.12*s*. The P wave and the T wave are generally longer than the QRS complex. Hence, in the proposed study, *s*_*m *_= 20 for MIT-BIH database and *s*_*m *_= 15 for QT database, were chosen. No calculation was performed at other scales since MMD operation does not cause drift of singular points across different scales.

For the detection of local maxima and minima, two thresholds *Th*_*R *_and *Th*_*f *_were used, which were adaptively computed from the histogram of the MMD transformed data. The two between-peak valleys in the histogram gave rise to the values of *Th*_*R *_and *Th*_*f*_*. Th*_*R*_was used for the detection of the local minima, which correspond to R peaks; *Th*_*f *_was used for the detection of the local minima, which correspond to other characteristic waves.

In any single ECG beat, the R peak, Q wave and S wave correspond to adjacent local minima in the morphological derivative-transformed signal. The onset and offset of the P wave correspond to local maxima adjacent to the Q wave. In the normal cases, the onset and offset of the T wave are local maxima adjacent to S wave. Otherwise, the T wave is judged as inverted. However, for other abnormal T waves, such as the biphasic T wave, the MMD detector may falsely detect the onset and offset of the T wave.

## Results and discussion

The proposed morphological approach for the characteristic wave detection in ECG signal was tested using the first ECG leads from the MIT-BIH arrhythmia database [21] and the QT database [22], which were developed with the aim to be benchmarking references for automated analysis of ECG. The MIT-BIH arrhythmia database contains 48 records (each 30 minutes long) with a sampling frequency of 360 *Hz*. The QT database is a mixed database with a sampling frequency of 250 *Hz*, which consists of 105 excerpts (each 15 minutes long) taken from other ECG databases, where, 15 from MIT-BIH Arrhythmia Database, 6 from the MIT-BIH ST Change Database, 13 from the MIT-BIH Supraventricular Arrhythmia Database, 10 from the MIT-BIH Normal Sinus Rhythm Database, 33 from the European ST-T Database, 24 from "sudden death" patients from BIH, and 4 records from the MIT-BIH Long-Term ECG Database.

**Figure 1:**
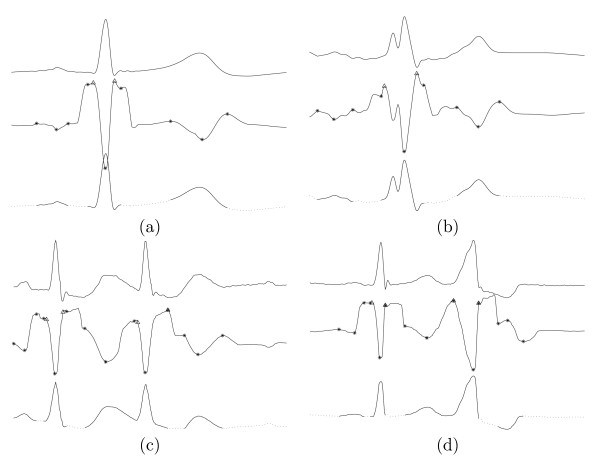
Results of characteristic wave detection for single ECG beat. The three plots in each subplot from top to bottom are: original ECG signal; the MMD transformed signal at scale 20 with the detected characteristic points marked; detected characteristic waves in solid line. (a) Normal ECG beat (b) LBBB (c) APC (d) PVC.

For each input ECG signal, the following procedures were performed: (i) signal preprocessing; (ii) multiscale morphological derivative transform; (iii) detection of local maxima and minima in morphological derivative-transformed signals; (iv) detection of characteristic waves in the original ECG signal. Some results using MMD detector for characteristic wave detection are given in Figure 1, where (a) is for normal ECG beat, (b) is for left bundle branch block (LBBB) ECG beat, (c) is for atrial premature contraction (APC), and (d) is for premature ventricular contraction (PVC). In each subfigure, the three plots from top to bottom are: the single ECG beat selected from the MIT-BIH database; the MMD transformed signal with marked points (the onset and offset of the R wave are marked with 'Δ'; other fiducial points, such as, the Q wave, R peak, S wave, as well as the onsets, offsets, the peaks of the P wave and T wave, are marked with '*'); the automatically detected characteristic waves are in solid line.

As shown in Figure 1, the characteristic waves in normal beat are observed to be well detected. For LBBB, in spite of the appearance of a sub-R peak, the boundaries of all waves are still well detected. In APC or PVC no preceding premature P wave appears. In addition, the position of the left 'Δ ' overlaps with the '*' because the onset of the preceding T wave is submerged in the QRS complex. The position of the right 'Δ' also overlaps with the '*' and the Q wave is judged to be missing.

**Figure 2:**
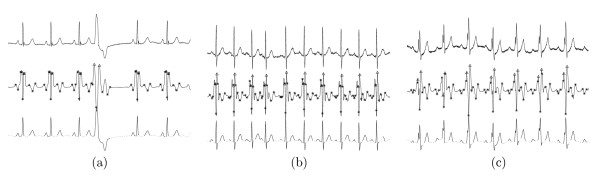
Detection results of ECG series, from top to bottom, they are: original ECG signal; MMD transformed signal with fiducial points marked; detected characteristic waves highlighted in bold. (a) ECG series with normal beats and PVC (b) ECG series with normal beats and APC (c) ECG series with LBBBs.

Figure 2 gives more results of characteristic wave detection in ECG signal series. It is obvious that all three characteristic waves (the QRS complex, the P wave, the T wave) in ECG time series with normal beats, APCs, and LBBB beats, are detected reliably. Even the onsets and offsets of inverted T waves in PVCs can be detected reliably.

More than 20000 annotated ECG beats randomly selected from MIT-BIH arrhythmia database were used to test the performance of the proposed MMD detector. An overall false detection rate of 0.35% was obtained for the QRS complex detection. In addition, more than 2500 annotated ECG beats randomly selected from the QT database were tested for evaluating the performance of the proposed MMD detector for ECG wave boundaries detection. In the QT database, a minimum of 30 beats from each of its 105 records had been manually annotated by one or more cardiologists. The annotation files were taken as reference for evaluating the performance of automated algorithms for detecting the onsets and offsets of the P wave, T wave, and the QRS complex during ECG analysis.

In order to quantify the performance of ECG characteristic wave detection by the proposed MMD technique, three parameters were used, which included the mean error (*m*) and standard deviation (*σ*) of the differences between the annotation results and the automated detection results, as well as Sensitivity (*Se*). *m *is used to determine how close the automated detection results are to the annotation results. *σ *gives an idea of the stability of detection. *Se *is defined as , for measuring the detection sensitivity, where *TP *is the number of true detections; *FN *is the number of manual annotations that are not registered in the automatic detections.

The statistical results for *m*, *σ*, and *Se*, for ECG fiducial points and characteristic waves detection by the proposed MMD technique were compared with the threshold-based detector (TD) [22] and the wavelet-based detector (WD) [23], and they are shown in Table 1. The accepted standard deviation tolerances from the measurements required by CSE were given in the last row of Table 1.

**Table 1:** Comparative Results of ECG characteristic wave detection

*Technique*	*Parameter*	*P*_*on*_	*P*_*off*_	*QRS*_*on*_	*QRS*_*off*_	*T_on_*	*T*_*off*_
MMD	*Se*(%)	97.2	94.8	100	100	99.8	99.6
	*m*(*ms*)	9.0	12.8	3.5	2.4	7.9	8.3
	*σ*(*ms*)	9.4	13.2	6.1	10.3	15.8	12.4
TD	*Se*(%)	96.2	97.0	99.9	99.9	98.8	98.9
	*m*(*ms*)	10.3	-5.7	-7.3	-3.6	23.3	18.7
	*σ*(*ms*)	14.1	13.6	10.9	10.7	28.3	29.8
WD	*Se*(%)	89.9	89.9	100	100	99.1	99.2
	*m*(*ms*)	13.0	5.4	4.5	0.8	-4.8	-8.9
	*σ*(*ms*)	12.7	11.9	7.7	8.7	13.5	18.8
CSE	*σ*(*ms*)	10.2	12.7	6.5	11.6	-	30.6

*Results on TD and WD are extracted from [10] and [11] for comparison purpose

The proposed MMD method works best for the QRS complex detection. Lower values of mean bias and standard deviation as well as higher value of detection sensitivity are observed. The average detection bias for the QRS's onset and offset are 3.5 *ms *and 2.4 *ms *respectively. The corresponding standard deviations are 6.1 *ms *and 10.3 *ms*, both of which are within the acceptable limits required by the CSE committee.

However the requirements can not be fully satisfied by the TD and WD methods although the mean bias obtained by the WD method is a little better than the results obtained by the MMD method. As for the P wave onset detection, only the MMD method can fulfill the CSE requirement while it fails for the P wave offset detection. The WD method performs better for P wave offset detection while worse for P wave onset detection compared with the MMD method. TD method fails to meet CSE requirements for both P wave onset and offset. As for the T wave offset detection, all three methods can satisfy the limit required by the CSE committee. Among them, the MMD and the WD methods perform much better than the TD method. The clinically important intervals for arrhythmia recognition, such as the PR interval (from the onset of the P wave to the onset of the R wave), the QT interval (from the Q wave to the offset of the T wave), are not related to the positions of the offset of the P wave and the onset of the T wave. Therefore, the weakness of the proposed MMD technique does not cause significant problem for arrhythmia recognition. The correlation coefficients between the results obtained by MMD method and those computed from annotation data are 0.9264 for PR interval, 0.9542 for QRS complex, and 0.9316 for QT interval.

The proposed MMD method for ECG wave boundary detection is well performed with reasonable mean bias and standard deviation values within the limits required by CSE, on over 79% of the records. In the QT database, best detection performance is observed for records from the MIT normal sinus rhythms database. Records with poor detection performance are mostly from the European ST-T database and the Supraventricular database, in which, low signal-to-noise ratio or non-homogeneous repolarization exists. In summary, it is concluded that the proposed MMD detector has acceptable performance comparable to those given by experts.

## Conclusion

In this paper, a new algorithm based on multiscale morphological derivative transform, called MMD detector, has been developed for fiducial point detection and applied for ECG wave boundary detection. The MMD detector could not only work for the QRS complex, but also the onsets and offsets of the characteristic waves. The standard deviations for important ECG characteristic wave detection obtained by the proposed MMD detector were within the limits required by the CSE committee. Furthermore, the statistical results obtained by the MMD detector were compared with those obtained by wavelet transform-based and adaptive thresholding-based techniques. In overall, better performance by the MMD technique was observed, considering that less empirical parameters were needed. Therefore we conclude that the proposed MMD method exhibits good potentials in the clinical applications for automated analysis of ECG signal.

## Competing interests

The author(s) declare that they have no competing interests.

## Authors' contributions

SY conceived the study, performed data analysis and drafted the manuscript. CKL and KSM guided the study, helped the analysis and interpretation of the results, and critically reviewed the manuscript. All authors read and approved the final script.

## Pre-publication history

The pre-publication history for this paper can be accessed here:


